# Comparing the biomechanical characteristics between squat and standing serves in female table tennis athletes

**DOI:** 10.7717/peerj.4760

**Published:** 2018-06-01

**Authors:** Changxiao Yu, Shirui Shao, Julien S. Baker, Yaodong Gu

**Affiliations:** 1 Faculty of Sports Science, Ningbo University, Ningbo, China; 2 School of Science and Sport, University of the West of Scotland, Hamilton, UK

**Keywords:** Relative loads, Kinematic chain, Lower limb, Center of pressure, Angular changing rate

## Abstract

**Background:**

The table tennis serve involves complex spatial movements combined with biomechanial characteristics. Although the differences in lower-limb biomechanial characteristics to a great extent influence the translational and spinning velocity of the ball when using the different styles of table tennis serve, few researchers have studied their mechanics. Therefore, the aim of this study was to investigate the differences in lower-limb activity between the squat and standing serves during a table tennis short serve.

**Methods:**

Ten advanced female table tennis participants performed a squat serve and standing serve in random order. A Vicon motion analysis system and a Novel Pedar insole plantar pressure measurement system were used to record kinematics and kinetics data, respectively.

**Results:**

Key findings from the study were that the squat serve not only showed significantly larger hip and knee flexion, as well as ankle dorsiflexion, it also showed significantly larger hip adduction and external knee rotation, with larger changing angular rate of the lower limb joints in the sagittal and the transverse planes when the two serving styles were compared. In addition, the force-time integral (FTI) was higher in the rear foot area for the standing serve.

**Discussion:**

The results demonstrated that the squat serve needs higher lower limb drive during a table tennis short serve compared with a standing serve. These biomechanical considerations may be beneficial for table tennis athletes and coaches as a method of optimizing performance characteristics during both competition and training.

## Introduction

Table tennis is a complex and asymmetric sport, and whose serve is the fundamental closed skill that requires active movement and accurate control. The ability to serve well in table tennis is a crucial part of the game. This enables the player to score quickly, and to gain an advantage. In addition, a powerful swing that transmits the moment of inertia to the ball with high speed, in the appropriate direction is vital for success. The table tennis serve is also an extremely important element that could provide a link to the next phase of play. A forceful lower limb drive is considered as the “starting point” of the kinetic chain at the serve point (Elliott, 2006). This could influence the qualities of batting (racquet and ball speed and a high degree of accuracy, etc.) (Girard et al., 2010; Girard, Micallef & Millet, 2005). High quality table tennis serves not only require greater upper limb co-ordination, but also need support from the lower limbs’ to provide accuracy and stability during competitions. However, despite obvious differences that exist between the two serve types related to lower limb contribution, coordinated motion patterns that produce better accuracy and faster connections with the next stage of play, are the greatest concern for both coaches and athletes.

Fu et al. (2016a) stated that high speed and heavy spin of the ball during the table tennis serve are two important scoring factors. Iino & Kojima (2009) reported that higher racket speed for advanced table tennis athletes mainly came from the lower trunk axial rotation on impact, which may contribute to increasing the translational and spinning velocity of the ball. The speed of a table tennis ball after it is impacted could reach 100 km/h (Xie, Teh & Qin, 2002). According to previous studies, the lower limb is the source of energy that could be transferred to the upper limb via sequential movements of the kinetic chain (Elliott, 2006; Qian et al., 2016). Moreover, impact is facilitated or attenuated from initial contact in different projectile sports (Sim & Kim, 2010; Bootsma & Van Wieringen, 1988; Franks, Weicker & Robertson, 1985; Coello et al., 2000). Initial correct contact is a complex motion that needs a period of swinging prior to impact (Müller & Abernethy, 2006; Bootsma & Van Wieringen, 1990). The control of movement patterns in the table tennis serve requires a coordinated sequence of body segment interactions, and the optimum activation of all the links has been defined as the “kinetic chain” (Girard, Micallef & Millet, 2005; Kibler & Van Der Meer, 2001). The coordinated activation of the sequence of events would be beneficial in producing optimal racquet positions, correct trajectory and required velocity on ball impact.

It has been well documented that serve efficiency, is related to upper-limb coordinated sequential activity, however, there is no systematic study attempting to explore the mechanisms, contributions and differences of the lower limbs between squat and standing serve styles in table tennis. Previous studies showed that the linear and angular momentum of the racket during striking motions are definitely related to lower limb drive (Elliott, 2006; Fu et al., 2016a; Wang et al., 2010; Lees, 2003). For energy generation and energy transference in the kinetic chain, the hip joint acts as the essential segmental link between the trunk and the leg. Qian et al. (2016) reported that increased hip flexion during the table tennis forehand loop may be a potential factor influencing the full-forward swing, which in turn could increase racket velocity. Iino & Kojima (2001) also emphasized that the hip joint had a vital effect on the trunk rotation in the tennis forehand stroke. Compared with novice servers, Kovacs & Ellenbecker (2011) found that elite servers have a more vigorous knee extension and a quicker knee extension velocity during the tennis serve. In addition, Reid, Elliott & Alderson (2008) reported an increased mean in extension range and peak angular velocity of the rear knee during the tennis serve. Mok et al. (2011) and Fong et al. (2012) stated that ankle motion was important in lower limb drive, but also indicated that ankle ligament sprain was the most common injury in sports.

Many studies have tried to investigate the kinematics of table tennis, comparing the relationship between different ball velocities and athletes of different performance levels (Bootsma & Van Wieringen, 1990; Marinovic, Iizuka & Freudenheim, 2004). However, as the origin of the kinetic chain, “Foot (shoe)-ground” biomechanical characteristics should be taken into account. To investigate the mechanical basis of the table tennis serve, both for better understanding the mechanisms and performance improvement, the ability to use a Novel Pedar insole plantar pressure measurement system is essential. Table tennis is a classic sport that typically needs upper limb, lower limb and abdominal simultaneous contractions to complete the stroke performance instantaneously. The “core area” of the body plays an important role in weight control that contributes to different ground reaction forces in each foot during the backswing and forward swing phases. Moreover, the movement of the center of pressure (COP) is a common parameter used to assess lower extremity function. Fu et al. (2016a) indicated that superior players possessed a better foot drive technique. They also observed a greater ability, in superior players, for foot motion control during investigations of COP trajectory during the performance of topspin forehand loops. Also, they noted that the COP displacement measured could be used to evaluate sports performance, for example, to assess increases in stability and accuracy. Charles, Smith & McLean (2013) and Richardson, Hughes & Mitchell (2012) revealed that advanced golfers showed less COP displacement than the novice player during golf putting or chipping.

We suggest that the high quality of table tennis serve, not only needs the integration of complex movement patterns of the upper limbs for appropriate racket angle, but also demands a stable and forceful lower limb base. Therefore, the purpose of this study was to identify the differences in lower limb kinematics and kinetics between squat and standing serves in table tennis. The results could help coaches improve their understanding of the two serving styles in table tennis and therefore help to improve athletic performance in training and competition. The hypothesis was that: (1) the total time would be similar when using squat and standing serves; (2) squat serve would show greater joint angles compared with standing serve at key technique events with larger changing angular rate of the lower limb joints during backswing; (3) standing serve would show different the force-time integral from squat serve mainly in the rear foot area.

## Methods

### Participants

Ten female advanced players from Ningbo University table tennis team volunteered to participate in the study. Participants were National Division I players (age: 21.6 ± 0.3 years; body mass: 54.2 ± 2.8 kg; height: 1.64 ± 0.03 m; training experience: 15.8 ± 1.7 years). All participants were right-handed and used their own racquets. All experimental data collection was performed at the same time of day. The training time of the table tennis team was arranged at afternoon from Monday to Friday, which included 1.5–2 h for technical practice everyday and physical training. The physical training was constituted by strength practice (two times), endurance exercise (one time) and speed practice (one or two times) for three times each week. The participants had no history of lower-limb injuries within six months prior to the test and reported no previous surgery or foot diseases. Participants provided written informed consent prior to commencement of the study. No participant received any payment for the study. This study was approved by the Ethics Committee of Ningbo University and the participants were informed of experimental procedures and requirements (RAGH 20161216).

### Experimental set-up

This study took place in Ningbo University table tennis training gymnasium. Prior to testing, each participant was given a standardized warm-up of 20 min within the experimental environment. Then participants practiced serves for 15 min with increasing speed. Finally, all participants executed randomly the squat serve or the standing serve (based on the judgement of their coaches). A ball machine was placed 1.2 m away from the participant’s court, and projected balls directly to the right of the table. Individual players played the shot and stroked the ball back, this implied one complete test. Each participant did twenty tests using the two types of table tennis serve, until 10 acceptable serves were accomplished for squat and standing serves, respectively. A total of 10 s of rest was arranged when one type of serve was finished. Before initiating serve action, participants maintained the neutral position for 5 s. The participants were requested to stroke their balls to a *R* = 15 cm target area bordering the net of the right serve box at match pace. This equipment did not influence serve motions.

### Instrumentation

Three-dimensional kinematic data was captured using an eight-camera Vicon motion analysis system (Oxford Metrics Ltd., Oxford, UK) with a frequency of 200 Hz. This measuring system has been used previously in kinematic analysis of sports such as running and walking (Mei et al., 2015; Shu et al., 2016; Fu et al., 2016b). All participant were asked to wear tight-fitting pants, and sixteen reflective markers (diameter: 14 mm) were adhered on the lower limbs. The marker locations included: anterior-superior iliac spine, posterior-superior iliac spine, lateral mid-thigh, lateral knee, lateral mid-shank, lateral malleolus, second metatarsal head and calcaneus of the lower limb. Kinetic data was recorded by a Novel Pedar insole plantar pressure measurement system (Novel GmbH, Munich, Germany) at 50 Hz. This equipment has been used previously in kinetic analysis for table tennis and tennis (Girard et al., 2010; Fu et al., 2016a; Qian et al., 2016). Measuring insoles were placed bilaterally inside the participants’ shoes (size 38–40), and the data recording was sampled through Bluetooth technical equipment. Kinematic and kinetic tests were conducted synchronously.

### Data processing

**Figure 1:**
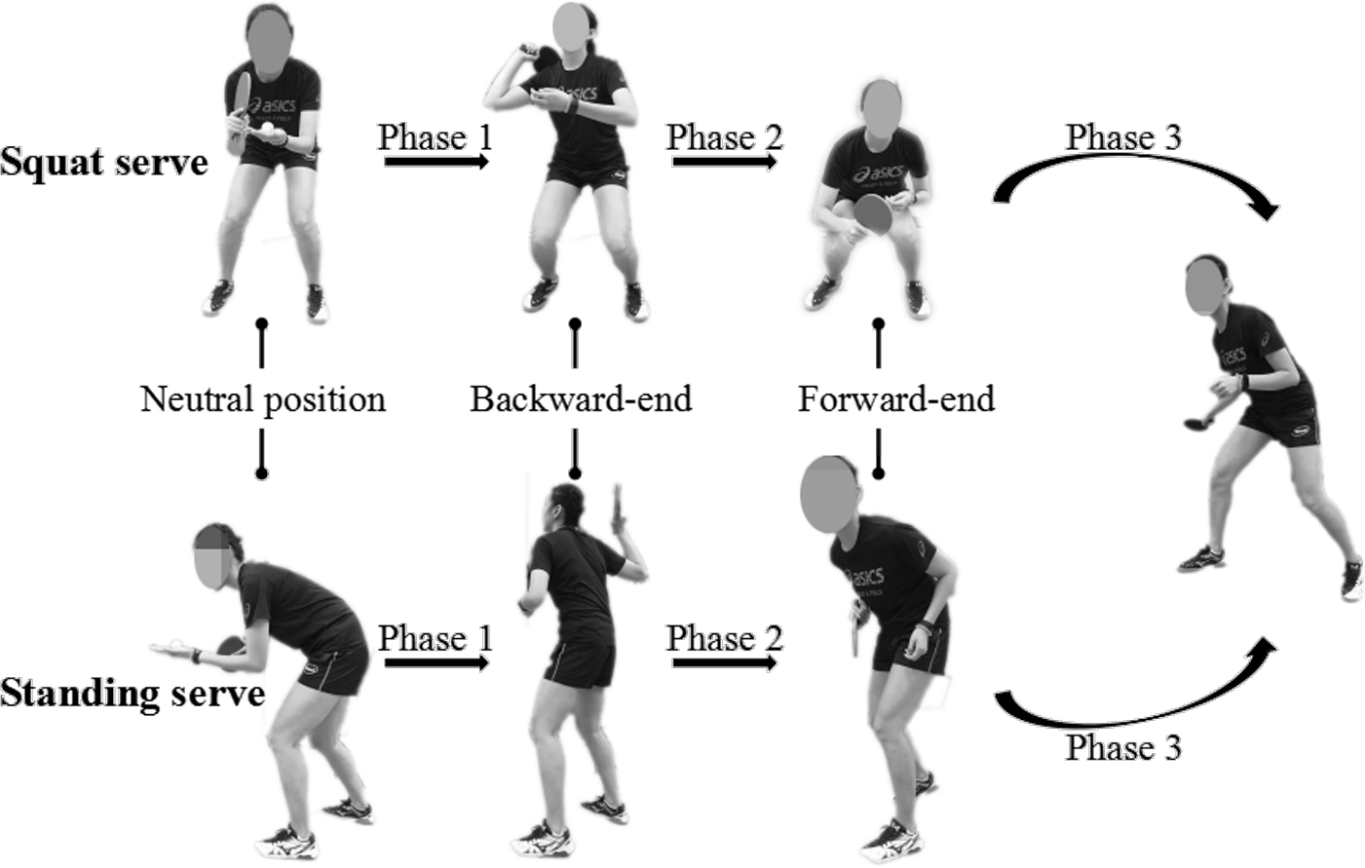
Four key events between squat and standing serves in table tennis.

A complete process was recorded from a neutral position to the initial stage of the next impact. This study divided one entire motion into three phases: from initiation to backward-end (phase 1), from backward-end to forward-end (phase 2), from forward-end to initial position of next impact (phase 3). In this study we selected three key events (ready position, RP; backward-end, BE; forward-end, FE) (Fig. 1) from the entire motion. Kinematic analyses were conducted on the following dependent variables: joint angles at RP, BE and FE events as well as the angular magnitude of two consecutive key events, joint angular changing rate between RP and BE, BE and FE, movement time from RP to BE, BE to FE, and FE to next start (NS) as well as the entire time between squat and standing serves in table tennis. Variables of the dominant lower limb such as the force-time integral in each foot area was calculated, and the COP was also measured. The plantar analysis of each dominant foot was performed using six separate “areas” of the foot: big toe (BT), lesser toes (LT), medial forefoot (MF), lateral forefoot (LF), midfoot (M) and rear foot (R).

### Statistical analysis

All statistical tests were performed using SPSS version 19.0 software (SPSS Inc., Chicago, IL, USA) for Windows. Shapiro–Wilk normality test was conducted that all cases were normally distributed. The kinematic and kinetic data were time-normalized to an entire motion cycle (100 data points). To examine the differences between the two types of table tennis serve, a paired-samples *t* test was taken for kinematic and kinetic data. The significance level for all tests was set at *p* < 0.05. The effect size was determined based on Cohen’s *d* which was used to compare the differences in the average of the two groups. Effect size (ES) represents Cohen‘s *d*. and is defined as small (≥0.2 and <0.5), medium (≥0.5 and <0.8) and large (≥0.8), respectively (Cohen, 1988). The statistical power of the analysis was calculated using NCSS-PASS 15.0 software.

## Results

The time of entire motions were 1.58 ± 0.05 s and 1.57 ± 0.06 s for squat and standing serves in table tennis (*p* = 0.604), respectively, with no significance. In addition, each phase of the two styles of table tennis serve were shown in Table 1, and there were no significant differences observed.

**Table 1:** Comparison of time in key phases between squat and standing serves, mean ± SD.

	Squat serve	Confidence intervals	Standing serve	Confidence intervals	*P*	Effect sizes (95% CL)	Power
Phase 1	0.51 ± 0.02	(0.50, 0.53)	0.50 ± 0.03	(0.47, 0.52)	0.103	0.19	0.89
Phase 2	0.20 ± 0.02	(0.19, 0.22)	0.21 ± 0.04	(0.18, 0.23)	0.776	0.16	0.35
Phase 3	0.86 ± 0.05	(0.83, 0.90)	0.87 ± 0.03	(0.84, 0.90)	0.914	0.12	0.35

**Figure 2:**
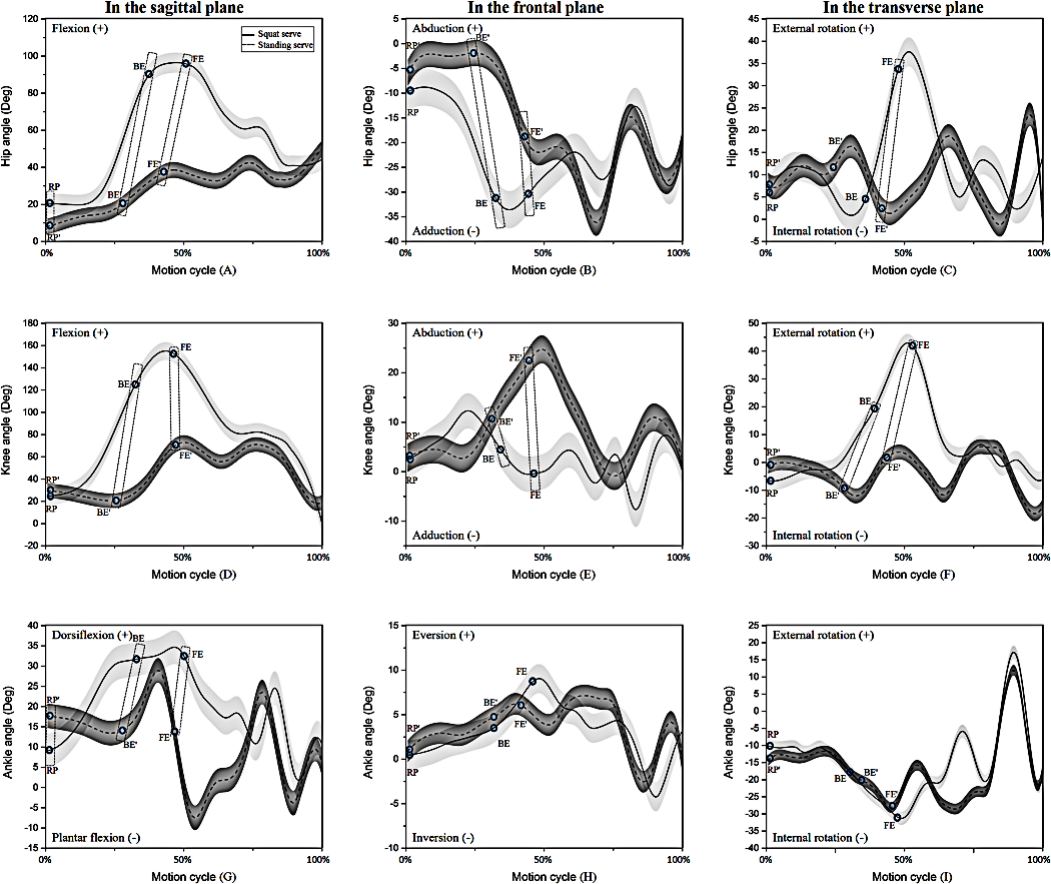
The trend of lower limb joints (A–C, hip; D–F, knee; G–I, ankle) during one motion cycle in three planes.

As shown in Fig. 2, there were significant differences in the joint angles at key events between squat and standing serves in the three planes. Compared with standing serve, the squat serve showed significantly larger hip and knee flexion as well as ankle dorsiflexion at BE and FE, it also illustrated significantly larger hip flexion but smaller ankle dorsiflexion at RP (Table 2). In the frontal plane, the squat serve displayed significantly larger hip adduction with smaller knee abduction than the standing serve at BE and FE (Table 3). For the squat serve, the hip and knee showed significantly larger external rotation at FE, in addition, at BE, the knee also illustrated significantly larger external rotation (Table 4).

**Table 2:** Comparison of joint angles at the moment of RP, BE and FE in the sagittal plane between squat and standing serves, mean ± SD.

	Squat serve	Confidence intervals	Standing serve	Confidence intervals	*P*	Effect sizes (95% CL)	Power
**Hip**							
RP	20.58 ± 4.76	(17.17, 23.98)	9.91 ± 0.78	(9.35, 10.46)	0.000^*^	0.84	1.00
BE	85.70 ± 0.86	(85.09, 86.32)	19.61 ± 1.02	(18.88, 20.34)	0.000^*^	1	1.00
FE	94.33 ± 1.56	(93.22, 95.45)	31.55 ± 3.19	(29.27, 33.83)	0.000^*^	1	1.00
**Knee**							
RP	25.67 ± 1.36	(24.70, 26.65)	27.11 ± 2.42	(25.38, 28.84)	0.146	0.34	0.99
BE	125.97 ± 5.57	(121.99, 129.95)	22.63 ± 0.96	(21.94, 23.32)	0.000^*^	1	1.00
FE	151.77 ± 2.13	(150.25, 153.29)	64.90 ± 2.43	(63.17, 66.64)	0.000^*^	1	1.00
**Ankle**							
RP	9.93 ± 1.53	(8.84, 11.03)	17.80 ± 1.85	(16.47, 19.12)	0.000^*^	0.92	1.00
BE	31.30 ± 1.36	(30.32, 32.27)	14.38 ± 1.13	(13.57, 15.19)	0.000^*^	0.99	1.00
FE	33.42 ± 1.17	(32.58, 34.26)	13.69 ± 1.14	(12.88, 14.51)	0.000^*^	0.99	1.00

**Notes.**

* indicates significant difference at the hip, knee and ankle (respectively) (*P* < 0.05).

**Table 3:** Comparison of joint angles at the moment of RP, BE and FE in the frontal plane between squat and standing serves, mean ± SD.

	Squat serve	Confidence intervals	Standing serve	Confidence intervals	*P*	Effect sizes (95% CL)	Power
**Hip**							
RP	−9.17 ± 0.87	(−9.80, −8.55)	−8.44 ± 0.92	(−9.10, −7.78)	0.085	0.38	1.00
BE	−32.18 ± 1.18	(−33.02, −31.33)	−3.88 ± 1.33	(−4.83, −2.93)	0.000^*^	1	1.00
FE	−31.95 ± 1.16	(−32.78, −31.12)	−19.94 ± 1.55	(−21.05, −18.83)	0.000^*^	0.97	1.00
**Knee**							
RP	2.62 ± 2.29	(0.98, 4.26)	4.24 ± 2.41	(2.52, 5.96)	0.132	0.33	1.00
BE	2.86 ± 2.72	(0.91, 4.81)	10.74 ± 1.29	(9.81, 11.66)	0.000^*^	0.88	1.00
FE	−0.01 ± 2.97	(−2.14, 2.12)	22.26 ± 2.78	(20.27, 24.25)	0.000^*^	0.97	1.00
**Ankle**							
RP	0.75 ± 1.52	(−0.33, 1.84)	1.34 ± 0.74	(0.81, 1.86)	0.240	0.24	0.67
BE	3.22 ± 1.04	(2.48, 3.97)	3.74 ± 0.82	(3.15, 4.32)	0.207	0.27	1.00
FE	7.36 ± 0.73	(6.84, 7.88)	6.70 ± 0.34	(6.46, 6.94)	0.064	0.50	1.00

**Notes.**

* indicates significant difference at the hip, knee and ankle (respectively) (*P* < 0.05).

**Table 4:** Comparison of joint angles at the moment of RP, BE and FE in the transverse plane between squat and standing serves, mean ± SD.

	Squat serve	Confidence intervals	Standing serve	Confidence intervals	*P*	Effect sizes (95% CL)	Power
**Hip**							
RP	7.08 ± 0.93	(6.42, 7.74)	7.69 ± 1.13	(6.88, 8.50)	0.188	0.28	1.00
BE	7.70 ± 0.81	(7.12, 8.28)	8.19 ± 1.14	(7.38, 9.01)	0.259	0.24	1.00
FE	34.33 ± 1.08	(33.55, 35.10)	1.99 ± 2.03	(0.53, 3.44)	0.000^*^	0.99	1.00
**Knee**							
RP	−5.19 ± 0.30	(−5.47, −4.91)	−4.87 ± 0.74	(−5.40, −4.34)	0.337	0.27	0.63
BE	19.05 ± 2.08	(17.56, 20.54)	−10.20 ± 0.74	(−10.73, −9.67)	0.000^*^	0.99	1.00
FE	41.26 ± 1.66	(40.08, 42.45)	−0.36 ± 1.16	(−1.19, 0.46)	0.000^*^	1	1.00
**Ankle**							
RP	−10.59 ± 1.65	(−11.78, −9.41)	−11.08 ± 1.25	(−11.97, −10.19)	0.456	0.17	0.97
BE	−18.49 ± 1.71	(−19.72, −17.27)	−19.32 ± 1.24	(−20.20, −18.44)	0.292	0.27	1.00
FE	−31.64 ± 1.73	(−32.88, −30.41)	−30.44 ± 1.31	(−31.37, −29.50)	0.120	0.36	1.00

**Notes.**

* indicates significant difference at the hip, knee and ankle (respectively) (*P* < 0.05).

**Figure 3:**
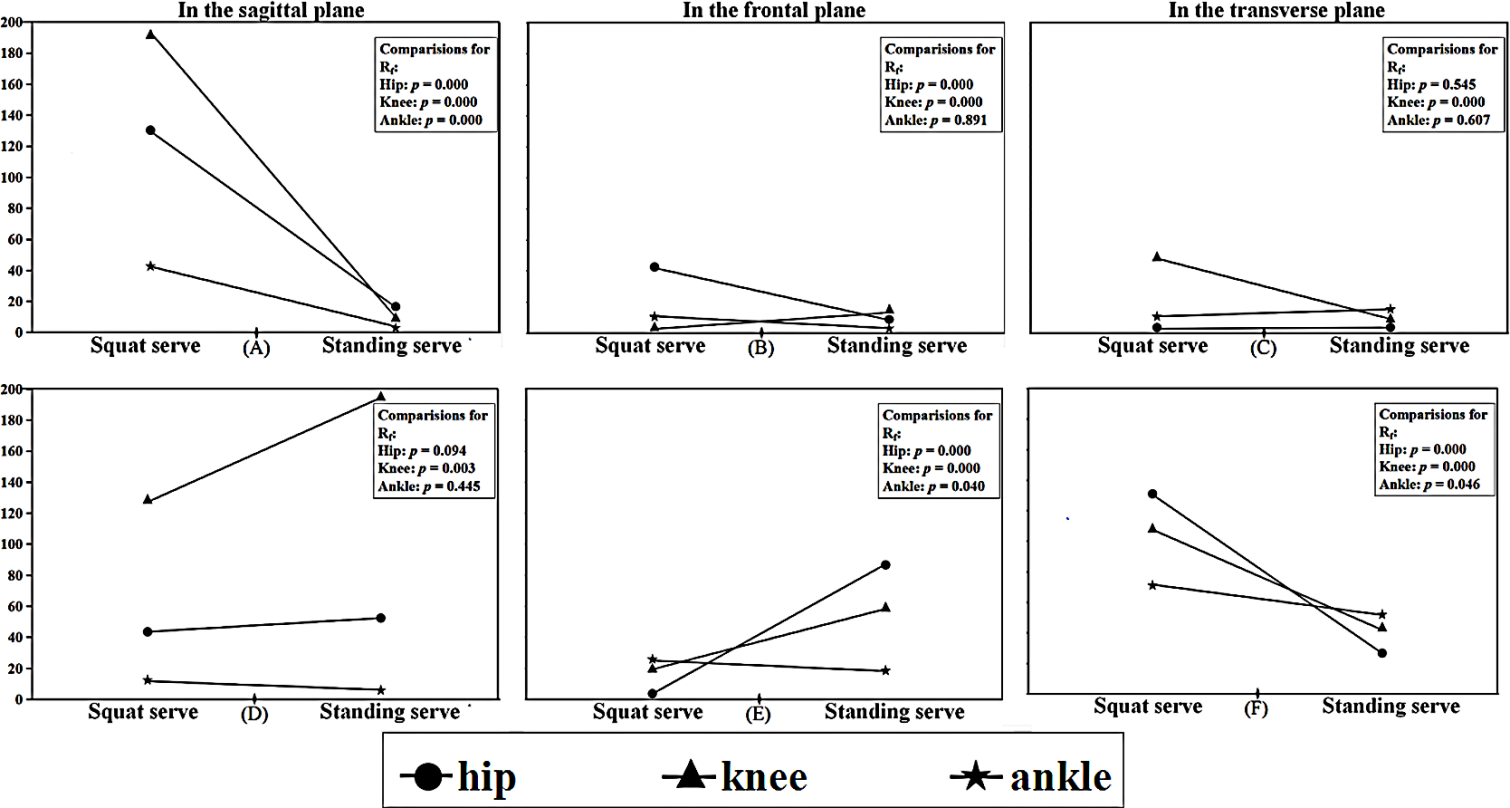
Angular changing rate of lower limb joints during phase 1 and phase 2 in three planes. (A) In the sagittal plane (B) in the frontal plane; (C) in the transverse plane; (D) in the sagittal plane; (E) in the frontal plane; (F) in the transverse plane.

Figure 3 revealed that *R*_*f*_ at the hip, knee and ankle for the squat serve during phase 1 was obviously larger in the sagittal plane (Fig. 3A) with larger *R*_*f*_ of the hip in the frontal plane (Fig. 3B) and larger *R*_*f*_ of the knee in the transverse plane (Fig. 3C), while it was slightly smaller at the knee than that of standing serve in the frontal plane (Fig. 3B). Moreover, for the squat serve, *R*_*f*_ was significantly larger at the lower limb in the transverse plane (Fig. 3F), while *R*_*f*_ at the hip and the knee were smaller with larger *R*_*f*_ of ankle in the frontal plane (Fig. 3E) during phase 2.

**Figure 4:**
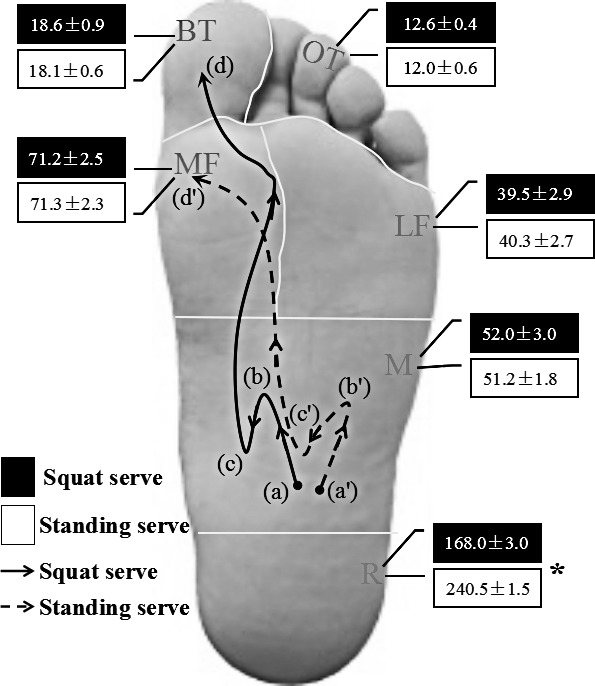
Mean and standard deviation relative loads and two typical COP movement trajectories for squat and standing serves in each of the six areas of interest. (a), (a′)—the original standing position; (b), (b′)—the start of the backswing; (c), (c′)—the end of the backswing or the start of the forward swing; (d), (d′)—the end of the forward swing. Big toe, BT; other toes, OT; medial forefoot, MF; lateral forefoot, LF; mid-foot, M; rear-foot, R.

Figure 4 shows that there were obvious differences in the COP trajectory shapes between the two types of serve. According to the COP movement characteristics, firstly the COP trajectories are evenly balanced in the mid-foot ((a), (a′) in Fig. 4), then move forward during initial phase 1 ((a)–(b) and (a′)–(b) in Fig. 4). It moved backward slightly in the backswing phase, the COP of squat and standing serves moved to the medial and lateral position, respectively ((b)–(c) and (b′)–(c′) in Fig. 4). After reversing along the inside of the foot the COP of the squat serve ended in the BT area. However, the standing serve ended in the MF area ((c)–(d) and (c′)–(d′) in Fig. 4). Concerning the force-time integral, it was significantly higher for the squat serve in the *R* area compared with standing serve (Fig. 4).

## Discussion

The primary focus of this study was to explore the variance in kinematics using the Vicon motion analysis system between squat and standing serves in table tennis. Also, using a Novel Pedar insole plantar pressure measurement system to speculate the plantar loads during the two types of table tennis serve. The main findings were that squat serve showed significantly larger lower-limb joint angles than the standing serve in the sagittal plane at BE and FE. In addition, there were significantly larger hip adduction and knee external rotation for the squat serve but smaller knee adduction at BE and FE. In the transverse plane, the squat serve also exhibited significantly larger hip external rotation at FE. Concerning the lower-limb joint *R*_*f*_, it was significantly larger for the squat serve than that of the standing serve in the sagittal plane during phase 1, and the hip in the frontal plane and knee in the transverse plane also showed the same condition, respectively. During phase 2, *R*_*f*_ at the lower-limb joint for the squat serve was significantly larger than that for the standing serve in the transverse plane. The ankle also showed the same condition but the hip and knee showed the opposite condition in the frontal plane. With respect to plantar force-time integral for the entire motion, the standing serve showed significantly higher values in the *R* area compared with the squat serve.

During the backswing phase, the lower-limb joint movement of the squat serve in three planes exhibited significantly larger hip and knee flexion as well as ankle dorsiflexion compared with standing serve. This suggests that larger ground reaction force can be generated for a table tennis player due to the lower center of gravity (COG) in conjunction with higher joint *R*_*f*_. Moreover, the results showed that the first slight backward movement for COP trajectory of both groups accounted for a rapid shift of plantar pressure to the *R* area during the backswing phase, and it also showed that the force-time integral was higher in the *R* area when using the standing serve style. Therefore, the standing serve may possess a fuller-backswing compared with the squat serve. In the present study, the squat serve showed significantly larger knee external rotation and hip adduction than the standing serve, and it also contributed to greater balance and was a potential factor to rapidly link the next stage of the kinetic chain. Based on the theory of the stretch-shortening cycle, that states prior stored elastic energy in a muscle–tendon stretching phase, could increase concentric movement (Elliott, 2006; Walshe, Wilson & Ettema, 1998), it can be inferred that the increased hip and knee flexion as well as the ankle dorsiflexion for squat serve at BE which could enhance muscle output of gluteus maximus and increase the racket speed at impact during the forward swing phase. Meanwhile, significant differences in the *R*_*f*_ of the ankle during the forward swing was also observed in the frontal and transverse planes. Compared with the standing serve, *R*_*f*_ at the ankle for the squat serve was clearly larger in phase 2. Moreover, the COP of the squat serve moved to the BT area, but that of standing serve moved to the MF area. This may suggest a more stable centre of mass shift for the squat serve during the forward swing.

Interestingly, compared with the standing serve, the squat serve showed significantly larger joint angles and angular changing rates of the lower limb during an entire motion. Seeley et al. (2011) reported that joint angular velocity is the vital factor to optimize energy transfer in the kinetic chain, which could increase the level of skill for athletes. Moreover, over-consuming energy could lead to a decrease in motion accuracy. Therefore, the squat serving requires higher physical quality particularly within the hip and knee. Due to table tennis being a multiple-set sport, elite athletes find it difficult to win by a single shot even within their skill set. This stresses the importance of improving physical quality and attributes to discover and develop marginal gains and therefore, improve performance.

Some significant limitations to the study should be noted. First, the study only recruited female athletes, there may be some differences between male and female performing a table tennis serve. Although the ten participants were granted with National Division I, it may limit the external validity at some degree. Further, the differences in the biomechanical characteristics between bilateral lower limb were not compared in the study. Future studies on serve in table tennis should pay attention to the relationship between the male and the female or between the right and left lower limb.

## Conclusions

This study is the first to investigate the differences in kinematics and kinetics during table tennis short serves between squat and standing serves. It provides a thorough understanding of lower limb joint movement patterns of advanced female table tennis players when using the two styles of different serve, which have important implications on sports performance enhancement. As the results of this study indicated, compared with the standing serve—although powerful lower limbs for the squat serve provide a stable base and higher hip, knee and ankle flexion—greater range of motion in conjunction with larger angular changing rate of the lower limbs which may be hard to maintain the control for the technical movements during a sustained period of exercise. For the standing serve, the force-time integral was higher on the *R* area than the squat serve, which may be helpful for greater racket speed at impact. Based on the study, the squat serve needs more flexible lower limb performance than standing serve for female athletes. Therefore, in order to master the particular serve technique in table tennis playing, the players should pay attention to the lower limb strength and flexibility exercises.

##  Supplemental Information

10.7717/peerj.4760/supp-1 Supplemental Information 1 Raw data exported from the different key phases between squat and standing serves applied for data analyses and preparation for the detailed investigation shown Table 1

10.7717/peerj.4760/supp-2 Supplemental Information 2 Raw data exported from the joint angles at the moment of RP, BE and FE between squat and standing serves during one motion cycle in three planes applied for data analyses and preparation for the detailed investigation

10.7717/peerj.4760/supp-3 Supplemental Information 3 Raw data exported from the angular changing rate of lower limb joints between squat and standing serves during phase 1 and phase 2 in three planes applied for data analyses and preparation for the detailed investigation

10.7717/peerj.4760/supp-4 Supplemental Information 4 Raw data exported from the mean and standard deviation the force-time integral and two typical COP movement trajectories for squat and standing serves in each of the six areas of interest applied for data analyses

## Additional Information and Declarations

### Data Availability

The following information was supplied regarding data availability: The raw data are provided as a Supplemental File.
